# Hydrodynamic study of freely swimming shark fish propulsion for marine vehicles using 2D particle image velocimetry

**DOI:** 10.1186/s40638-016-0036-0

**Published:** 2016-04-06

**Authors:** Mannam Naga Praveen Babu, J. M. Mallikarjuna, P. Krishnankutty

**Affiliations:** Department of Ocean Engineering, Indian Institute of Technology Madras, Chennai, 600 036 India; Department of Mechanical Engineering, Indian Institute of Technology Madras, Chennai, 600 036 India

**Keywords:** Carangiform swimming, Caudal fin locomotion, Flow visualization, Propulsor hydrodynamics, Particle image velocimetry, Pectoral fins, Reverse von Karman vortex street, Wake

## Abstract

Two-dimensional velocity fields around a freely swimming freshwater black shark fish in longitudinal (XZ) plane and transverse (YZ) plane are measured using digital particle image velocimetry (DPIV). By transferring momentum to the fluid, fishes generate thrust. Thrust is generated not only by its caudal fin, but also using pectoral and anal fins, the contribution of which depends on the fish’s morphology and swimming movements. These fins also act as roll and pitch stabilizers for the swimming fish. In this paper, studies are performed on the flow induced by fins of freely swimming undulatory carangiform swimming fish (freshwater black shark, *L* = 26 cm) by an experimental hydrodynamic approach based on quantitative flow visualization technique. We used 2D PIV to visualize water flow pattern in the wake of the caudal, pectoral and anal fins of swimming fish at a speed of 0.5–1.5 times of body length per second. The kinematic analysis and pressure distribution of carangiform fish are presented here. The fish body and fin undulations create circular flow patterns (vortices) that travel along with the body waves and change the flow around its tail to increase the swimming efficiency. The wake of different fins of the swimming fish consists of two counter-rotating vortices about the mean path of fish motion. These wakes resemble like reverse von Karman vortex street which is nothing but a thrust-producing wake. The velocity vectors around a C-start (a straight swimming fish bends into C-shape) maneuvering fish are also discussed in this paper. Studying flows around flapping fins will contribute to design of bioinspired propulsors for marine vehicles.

## Background

**Fig. 1:**
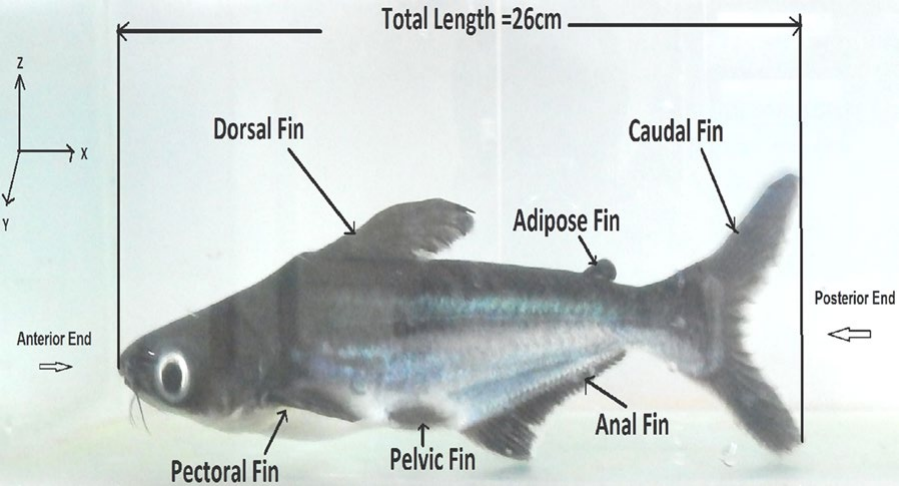
Freely swimming fresh water shark fish

**Fig. 2:**
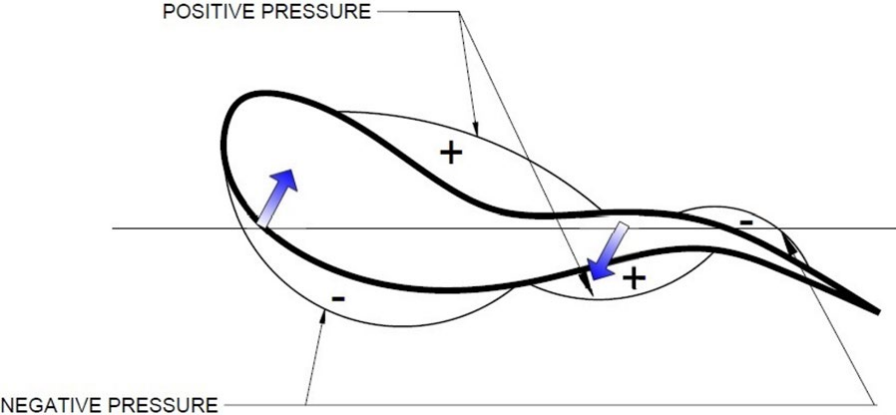
Pressure distribution around a swimming fish

**Fig. 3:**
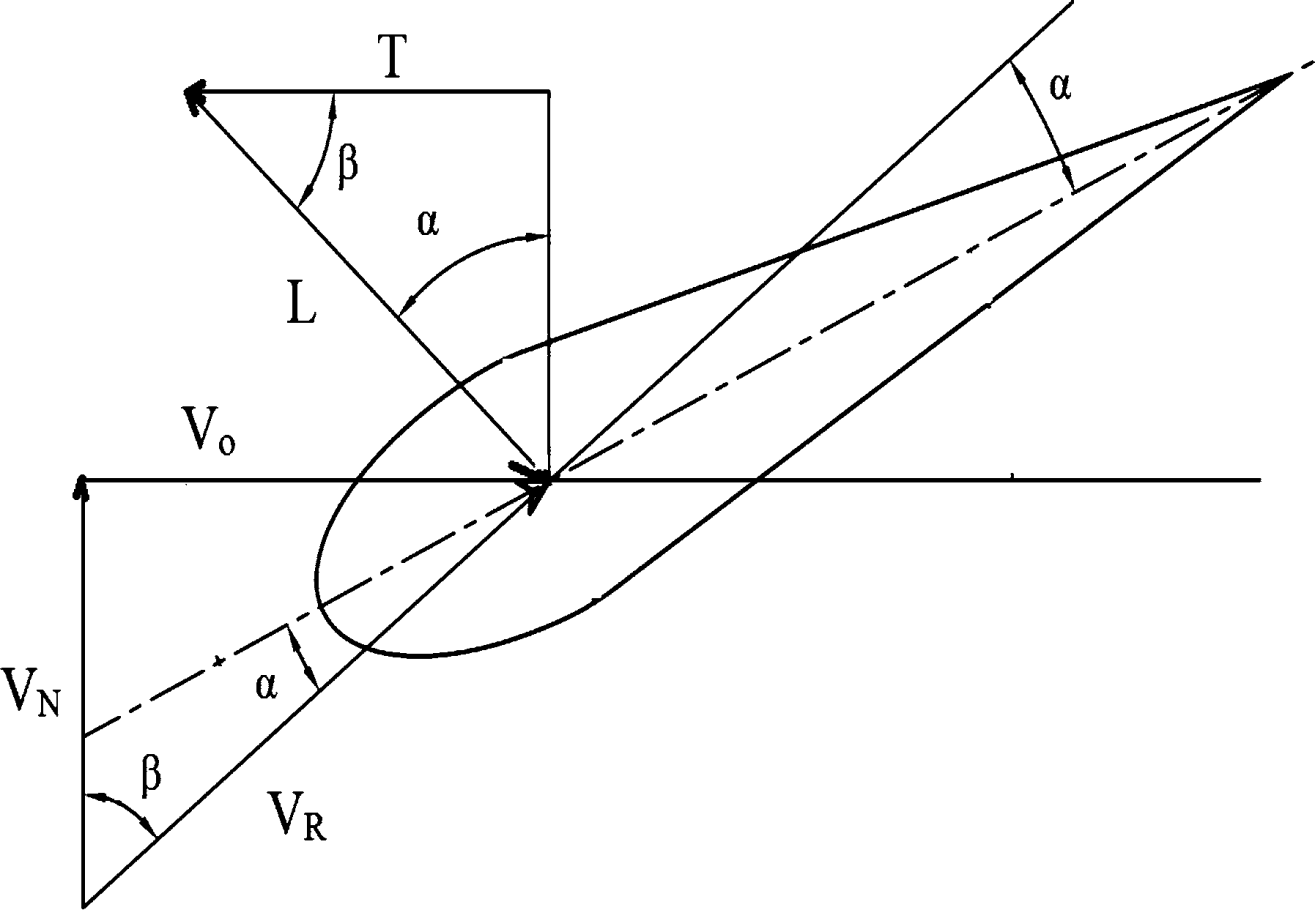
Velocity diagram of caudal fin

Aquatic animal propulsors are classified into lift-based (e.g., penguins, turtle forelimb propulsion and aerial birds), undulation (e.g., fishes, eels), drag-based (e.g., duck paddling) and jet mode (e.g., jelly fish, squids). Fishes use a combination of lift-based and undulating modes mainly using its undulating body, pectoral and caudal fins to achieve propulsive forces. Fishes also generate thrust by using its tail fin, paired fins and its body. Certain combinations of flapping motions and angles of body achieve greater speed and better maneuvering capabilities. Flapping foil propulsion systems, resembling fish fin propulsion mode, are found to be much more efficient than the conventional screw propellers [1, 2]. The application of fish propulsion to water crafts is found to have higher propulsive efficiency, better maneuvering capabilities, less vibrations, low emissions and more eco-friendly. Biological aquatic animal locomotion, its mechanism and their successful application to marine vehicles are being studied by different researchers. Muller [3] used 2D PIV to visualize the flow around the aquatic animals and to demonstrate the creation of vorticity and their contribution to thrust generation. Muller et al. [4] studied the water velocity near fish body using PIV and described the wake mechanism behind it. Drucker and Lauder [5] studied the bluegill sunfish pectoral fins 3D wake structures using PIV. Sakakibara et al. [6] used stereoscopic PIV for capturing three components of velocity distribution on live goldfish along with particle tracking velocity in order to determine spatial velocity, acceleration and vorticity. Past researchers [7–18] carried out experiments on hydrodynamic studies of fish locomotion as well as maneuvering by using PIV system. In the present study, a shark fish which belongs to the sub-carangiform is kept in a glass tank (Fig. 1) and the water particle kinematics around its tail and fins are observed using a two-dimensional PIV system while the fish try to swim forward. In sub-carangiform of locomotion, last one-third aft length of body muscle is used for generating thrust in addition to its caudal fin, whereas thunniform fishes’ the caudal peduncle and tail fin are responsible for thrust production. The sub-carangiform fishes can move its caudal fin at a higher amplitude compared with form of fishes, resulting better thrust generation. That is the reason for choosing this form of fish for the present study. It moves forward by flapping its caudal fin and body undulation. The pressure distribution around the body and the caudal fin is shown in Fig. 2. There are positive and negative pressure regions along the body. The fluctuations of these pressure distributions result in a propulsive force, pushing the fish forward. The shape of the caudal fin reduces the amount of displaced water during oscillation of tail fin, thereby reducing turbulence and frictional drag on the body without the loss of propulsive power. The velocity diagram of sub-carangiform fish caudal fin is shown in Fig. 3. In sub-carangiform swimming fish, the thrust is developed by the rear part of the body and the tail fin. The thrust generated by the tail fin is given by Eq. (1). The caudal fin is moving normal to free-stream velocity, *V*_o_, with a transverse (sway) velocity equal to *V*_N_. It is possible for caudal fin to attain a thrust component which provides a forward propelling force. The rotational component (yaw) is not considered in this case. The above equation is the simplest case of pure translation motion normal to a free stream *V*_o_ [19].

1 $$T = \left( {\frac{1}{2}} \right)\rho V_{\text{R}}^{2 } \left( {\frac{{{\text{d}}C_{L} }}{{{\text{d}} \propto }}} \right) S\alpha \cos \beta$$

where

2 $$\beta = \cos^{ - 1} \left( {\frac{{V_{\text{N}} }}{{V_{\text{R}} }}} \right)$$

where *ρ* represents density of fluid in kg/m^3^, *V*_R_ is resultant velocity in m/s, *S* is surface area of fin in m^2^, *α* is angle of attack in (rad) and (d*C*_*L*_/d*α*) is slope of lift curve for caudal fin.

In the present study, flow visualization experiments are carried out to visualize the flow pattern around the caudal, pectoral, anal and dorsal fins of a freely swimming fish using two-dimensional (2D) particle Image velocimetry (PIV) system.

## Methods

Freshwater black shark (Labeo chrysophekadion) with a body length of 26 cm is used for the present experimental study. The fish is placed inside a glass tank of size *L* × *B* × *D* = 75 cm × 29 cm × 37 cm, with water level at 28 cm, and it is allowed to swim freely in the tank. The fish swims across the tank length, and the PIV measurement is taken at the steady phase of its movement, which is observed to be in the middle one-third portion of the tank. In this experiment, the laser pulse is operated continuously and fish will always cross this laser plane in multiple times with same time interval. Then, a range of images is selected for processing velocity fields. From the visual observations, based on the recorded video, the Strouhal number of the freely swimming shark fish used in this experiment is approximately 0.23, where the tail fin oscillation frequency is 0.6 Hz, amplitude is 0.1 m and the fish swimming speed is 0.26 m/s.

## Experimental setup

Two-dimensional PIV technique is used to study the flow around a swimming fish. This helps in clearly understanding the instantaneous velocity vector fields of the flow field around the fish. It is a non-intrusive experimental technique which can measure the whole flow field with high spatial and temporal resolution at any instant.

The PIV technique involves the introduction of tiny particles called ‘seeder particles’ into the fluid path. The size and density of seeder particles are chosen such that they follow the flow path faithfully at all operating conditions. Hollow glass spheres with a mean diameter of 10 µm are used as the tracer particles. The seeding particles in the plane of interest are illuminated by a laser sheet of appropriate thickness 0.5–2.5 mm. Two images (an image pair) of the illuminated flow field are obtained within a separation time ‘∆*t*’ by means of high-resolution camera. The displacement of the tracer particles during the time interval ‘∆*t*’ gives velocity of the fluid particle. The experiments are performed at three different time intervals, Δ*t* = 300, 620 and 900 ms. If the Δ*t* is less than 300 ms, no swirl of velocity vectors is observed. Then, the Δ*t* gradually increases from 300 to 900 ms, and velocity fields around the fish body are observed. The Reynolds number (*Re*) of swimming fish is in the range of 10^5^, and at low *Re* number, the 2D velocity fields do not affect.

**Fig. 4:**
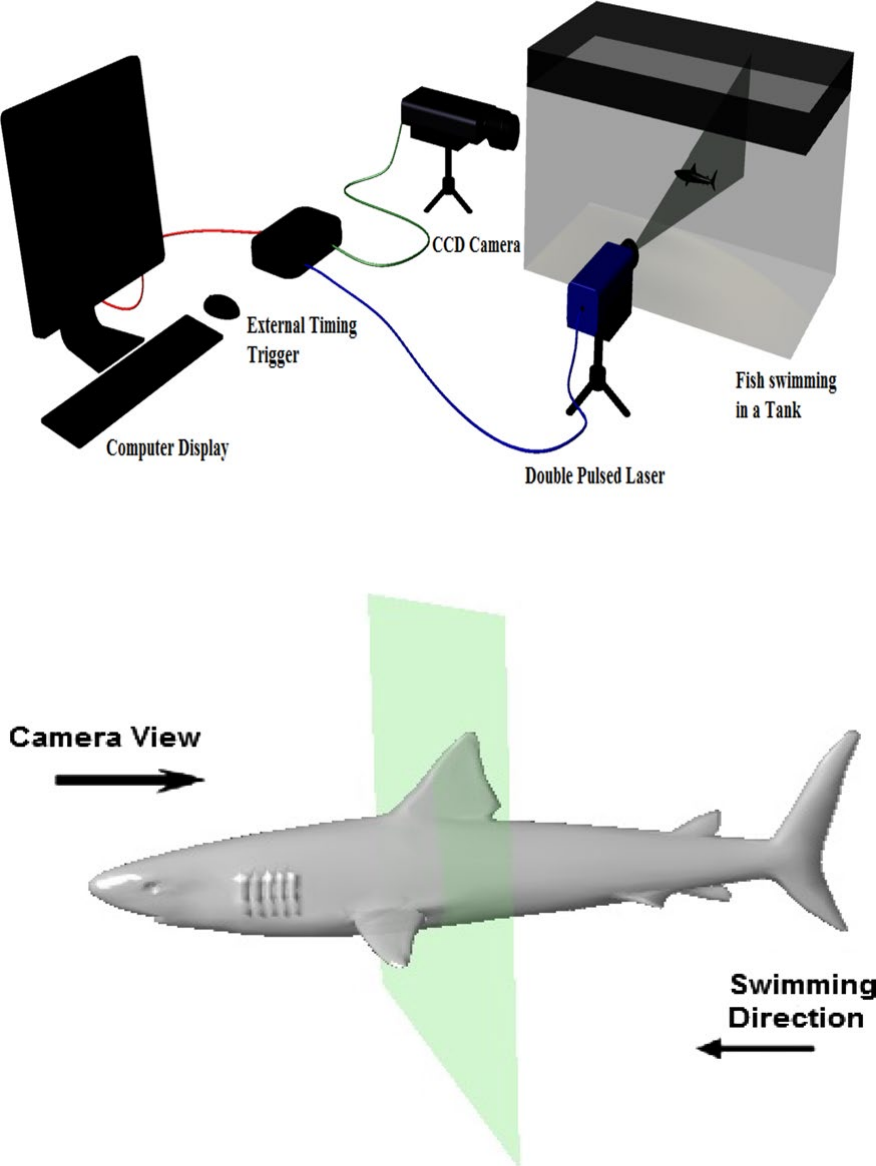
PIV system arrangement

**Fig. 5:**
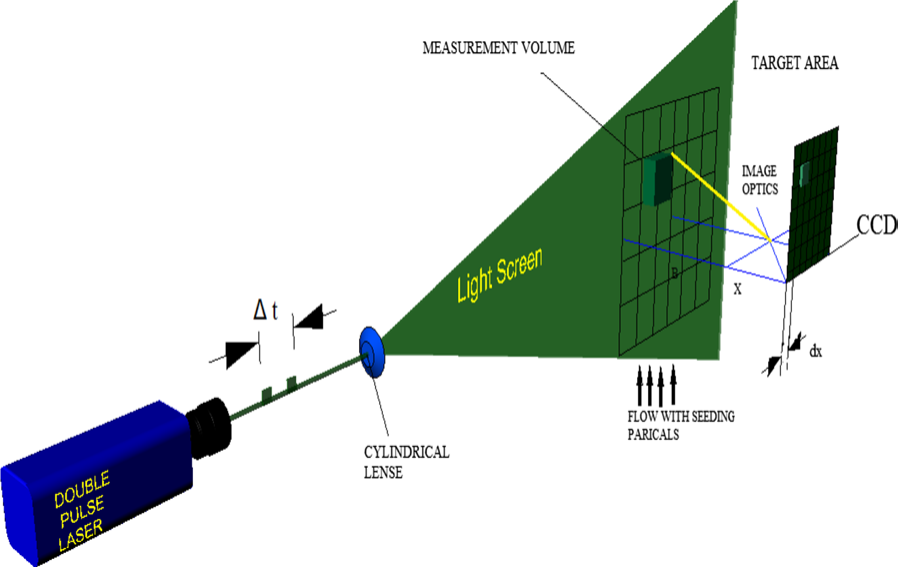
PIV system measurement scheme

The PIV setup used in the present study is shown in Fig. 4. The PIV system used in this work consists of (1) a double-pulsed Nd-YAG (neodymium-doped yttrium aluminum garnet) laser with 200 mj/pulse energy at 532 nm wavelength, (2) a charge-coupled device (CCD) camera with a 2048 by 2048 pixels and an image capturing speed of 14 frames per second (fps), (3) a set of laser and a camera controllers and (4) a data acquisition system. The laser sheet is aligned with the longitudinal vertical (XZ) and transverse (YZ) planes. The camera is positioned in front of the test section at 90° to the laser sheet (see Fig. 4). The size of seeding particles is very important in obtaining proper images. The particles should scatter enough light, and too large particles may not follow the flow path. Measurement of the velocity field using PIV is based on the ability of the system to accurately record and measure the positions of small traces suspended in flow as function of time. The PIV system measurement scheme is shown in Fig. 5.

In PIV measurement scheme, the images are divided into a number of small sections called interrogation windows or regions. The corresponding interrogation regions in frame 1 and 2 are correlated using cross-correlation method. The maximum of the correlation corresponds to the displacement of the particles in interrogation window. The displacement gives the vector length and direction in interrogation zones. Small interrogation windows give more vectors but contain less particles. The main advantage of the cross-correlation approach is displacement that can be obtained with directional ambiguity.

**Fig. 6:**
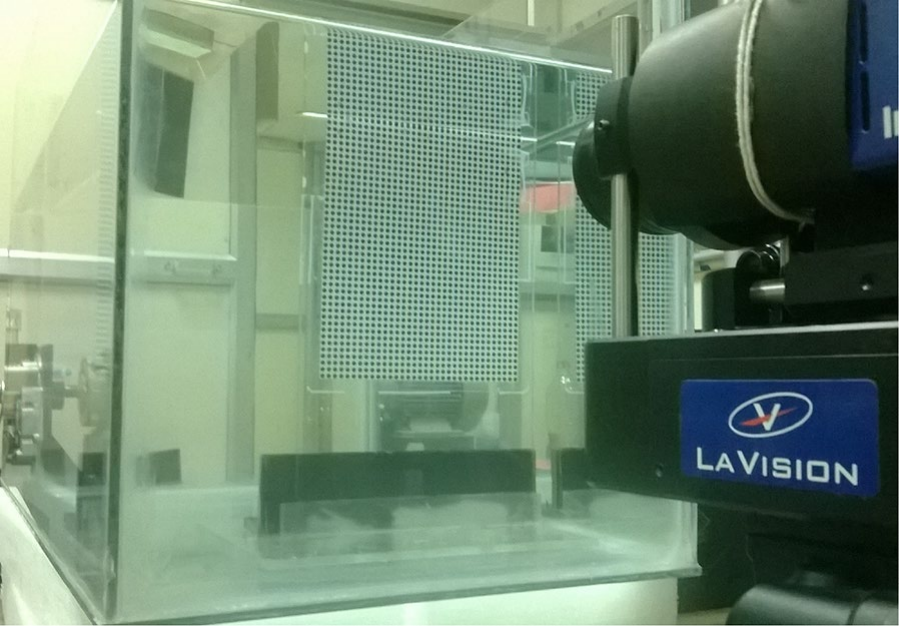
Calibration plate

In the experimental setup, care should be taken to make the laser sheet, camera axis and test object lie in the same plane. The laser sheet should be aligned perfectly vertical to the calibration plate (Fig. 6). In PIV calibration, the images obtained are focused and the scale factor (calibration constant) necessary for further processing of the images is obtained. This calibration plate is placed parallel to the light sheet and approximately in the middle of calibration sheet. The sheet consists of a grid of dots with a large central dot surrounded by four small dots. The distance between two dots on the calibration sheet is 5 mm. Images are captured on the calibration sheet when it is placed in the light sheet. These images are analyzed by the computer software ‘Davis’ [20], and the scale factor is obtained by comparing the apparent distance between the dots provided by CCD camera and the actual distance between the dots of 5 mm. Once the images are captured, the camera is focused on the sheet such that all dots appear sharp.

The velocity fields obtained by PIV are used to determine the pressure fields. The pressure and velocity are linked by the Navier–Stokes (NS) equations, and the pressure can be measured indirectly by measuring the velocity field. There exist two methods to measure the pressure field indirectly. The first method is direct spatial integration of the momentum equations [21, 22]. The second method is solving a Poisson equation for the pressure field [23]. The present study does not include the comparison aspects of velocity field to pressure field.

## PIV results and discussion

Fishes generate propulsive forces, are able to maneuver rapidly and stabilize its body motions using its fins such as pectoral, dorsal, pelvic, anal and caudal fins (see Fig. 1). By using its fins, fishes can control roll, pitch and yaw motions. The paired pectoral fins (one on each side) are used for maneuvering as well as for instantaneous stopping (braking) [24]. The median dorsal fins act as keels, used for directional stability and to prevent from spinning or rolling. Pelvic fins and anal fins are used as stabilizers. Caudal fin is used for propulsion, maneuvering and braking. The flow visualization experiments are carried out on a freely swimming sub-carangiform mode shark fish in longitudinal vertical (XZ) plane and transverse (YZ) plane by using two-dimensional particle image velocimetry.

**Fig. 7:**
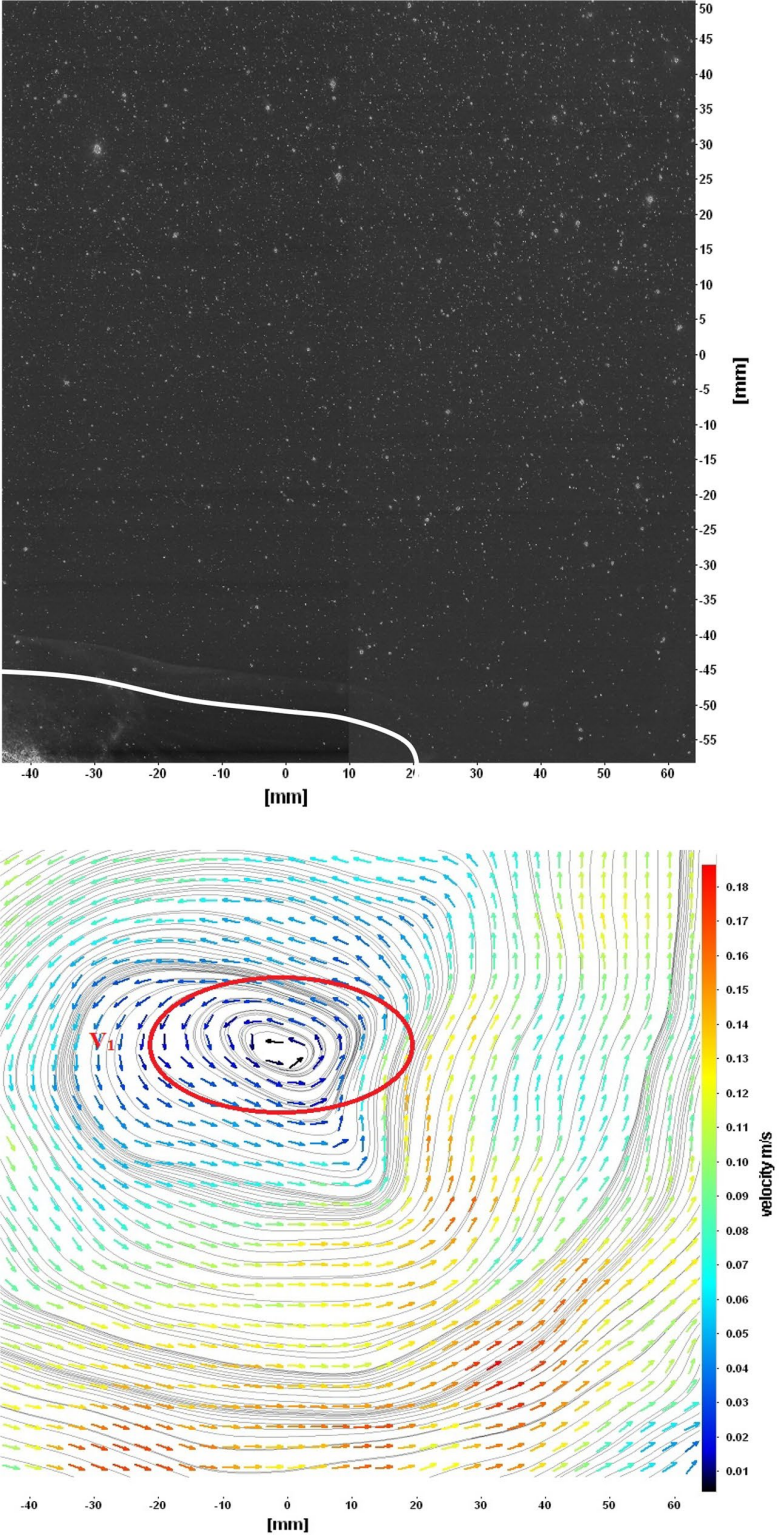
Raw image and velocity vector field around caudal fin at Δ*t* = 900 ms

**Fig. 8:**
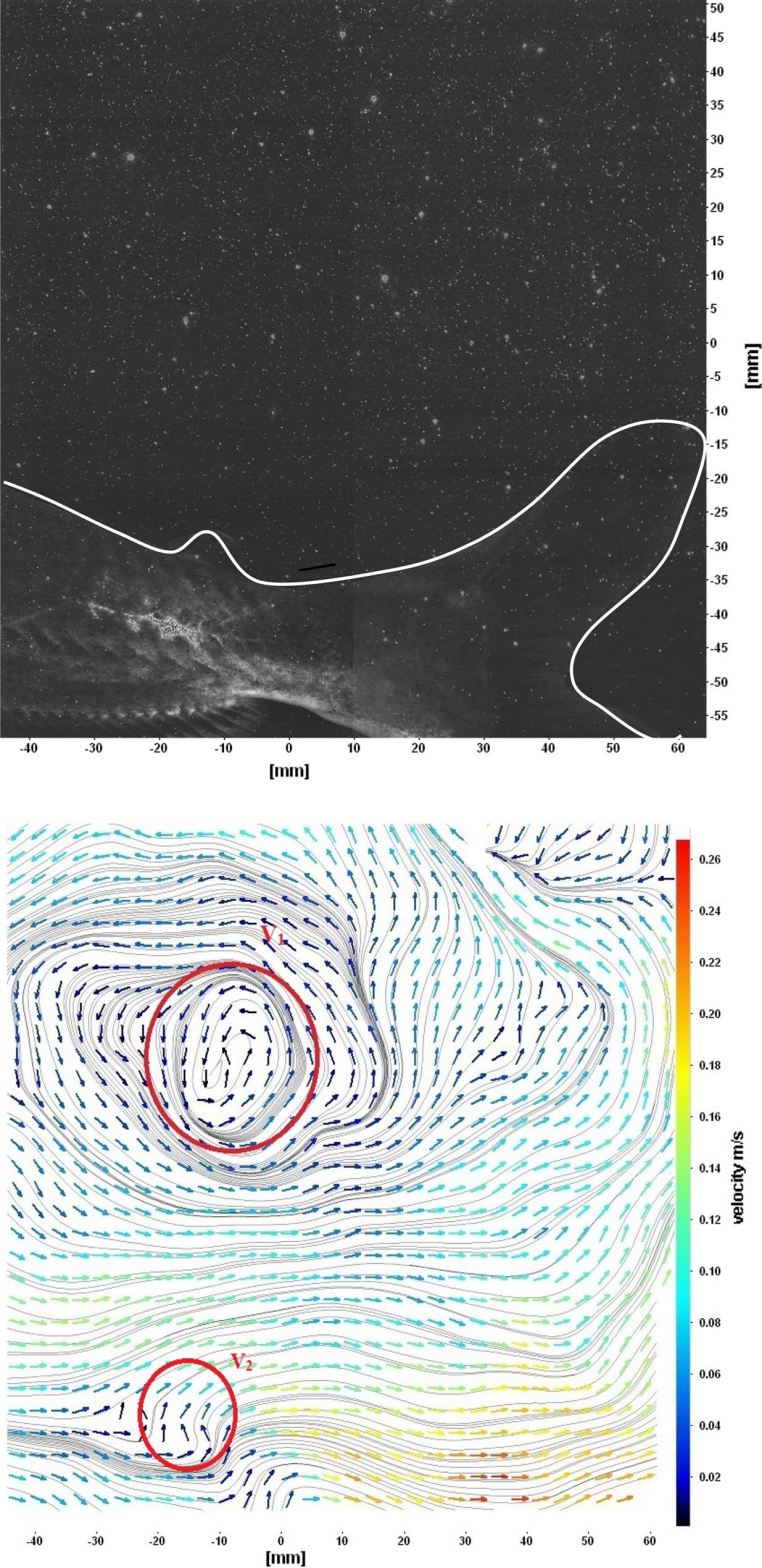
Raw image and velocity vector field around adipose (*upper one*) and anal (*below one*) fins at Δ*t* = 900 ms

**Fig. 9:**
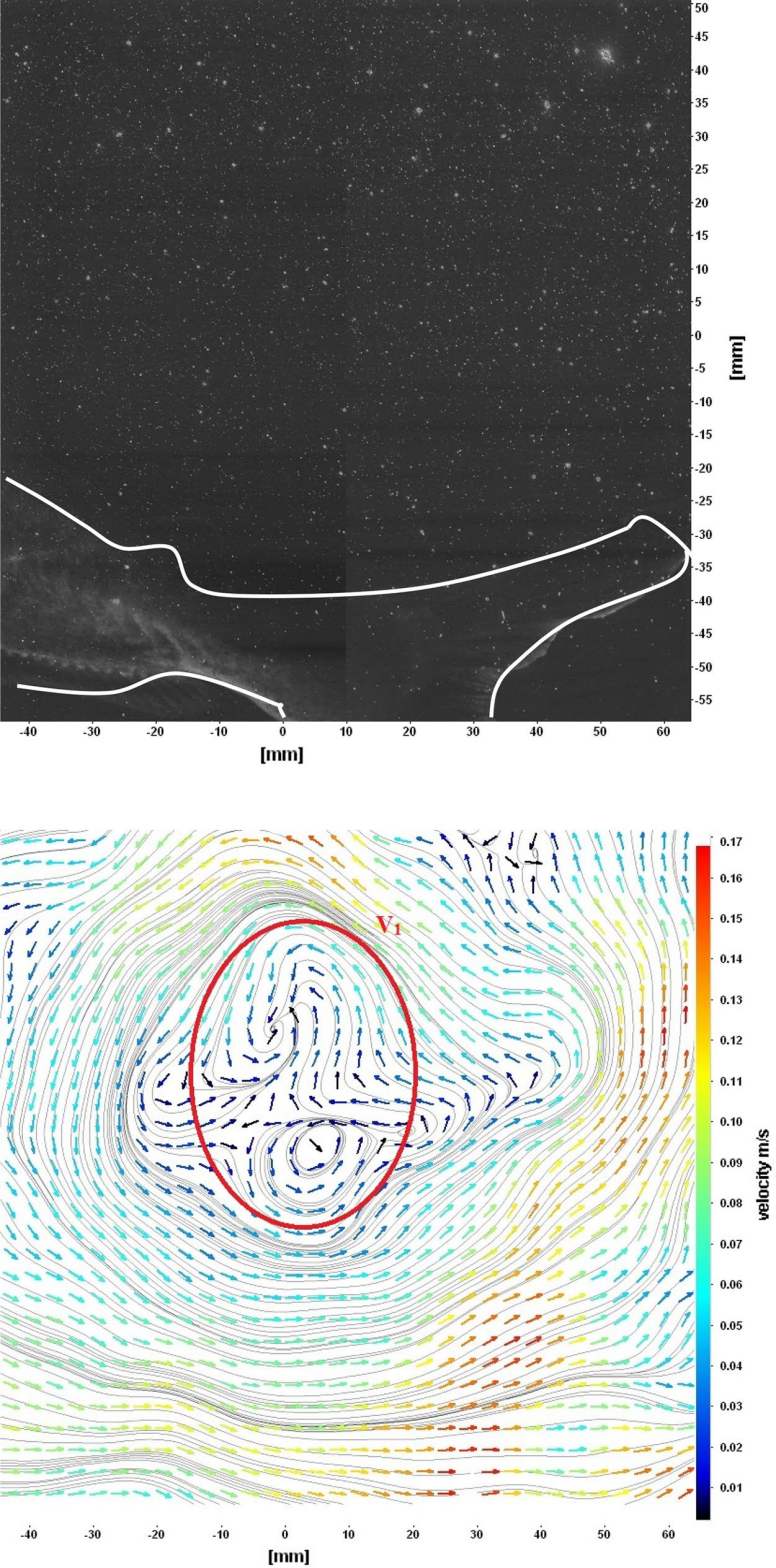
Consecutive raw image and velocity vector field around adipose and anal fins at Δ*t* = 900 ms

**Fig. 10:**
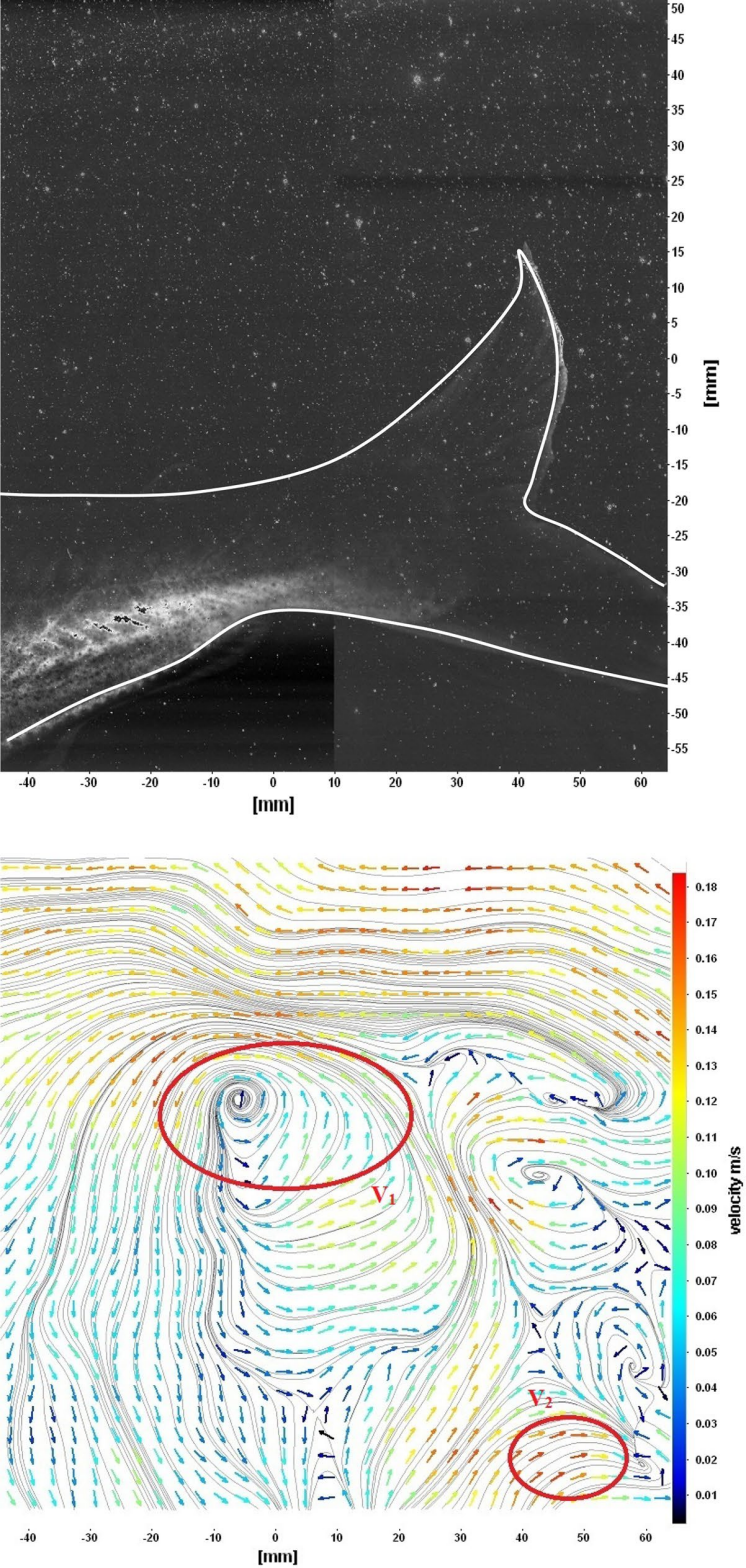
Raw image and velocity vector field around adipose and anal fins at Δ*t* = 300 ms

**Fig. 11:**
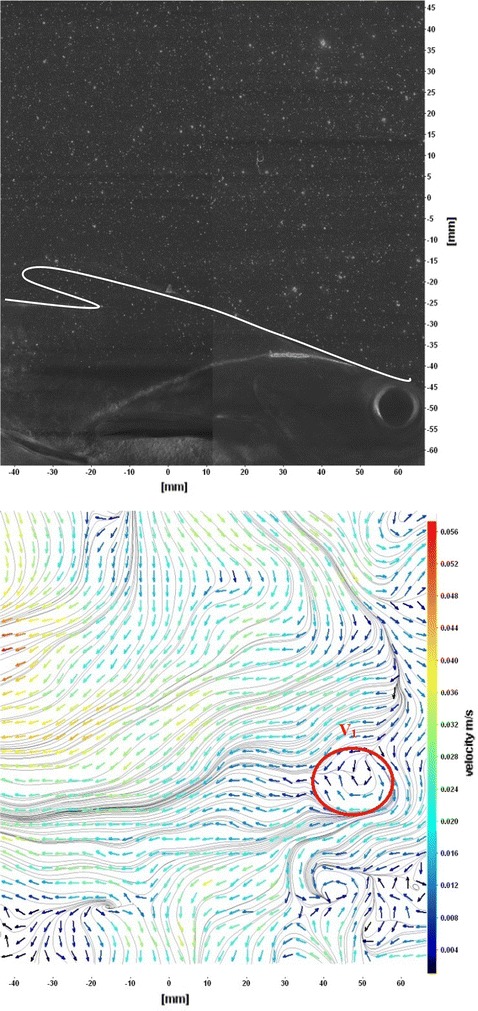
Raw image and velocity vector field around front (head) portion of fish

**Fig. 12:**
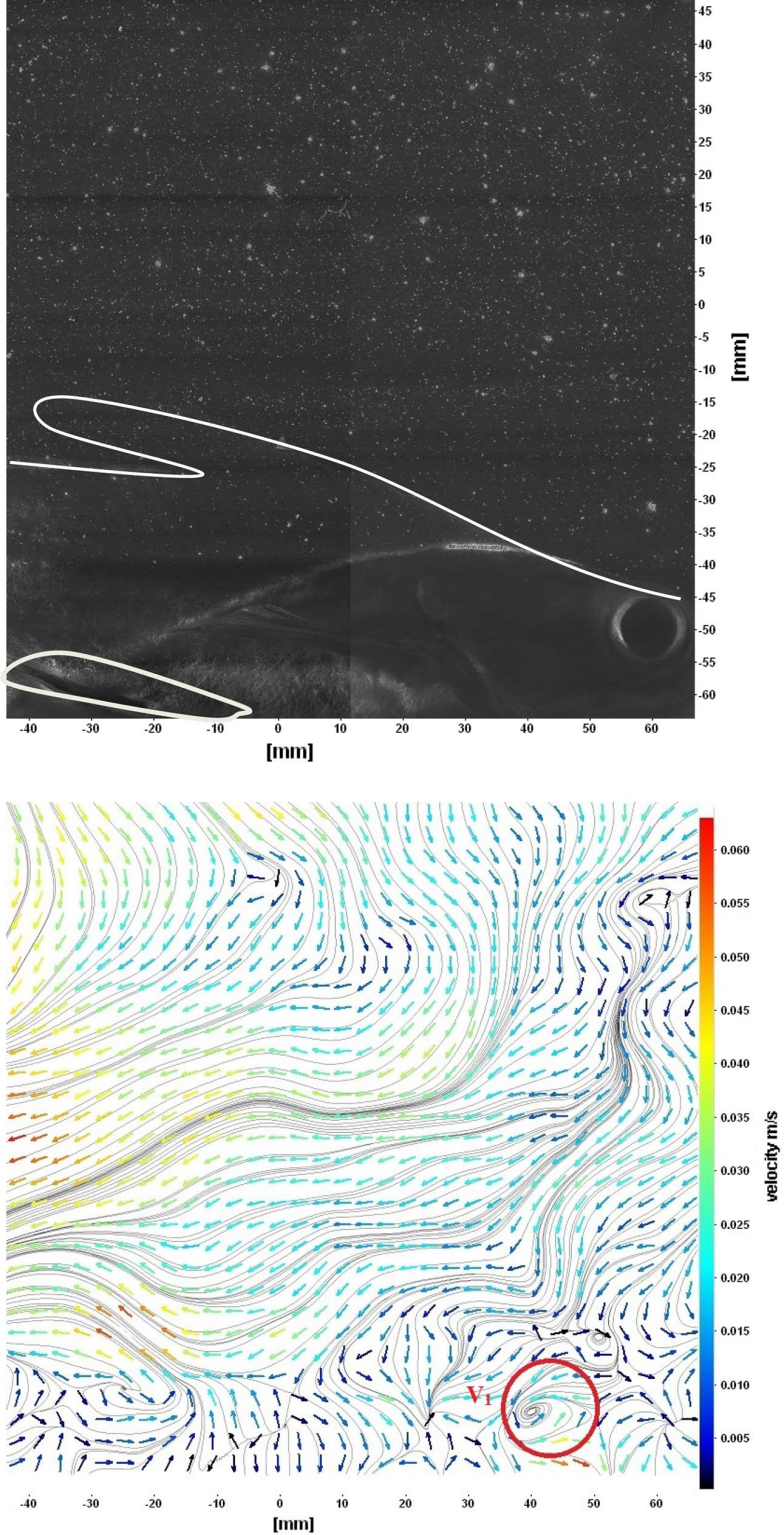
Raw image and velocity vector field around pectoral fins

**Fig. 13:**
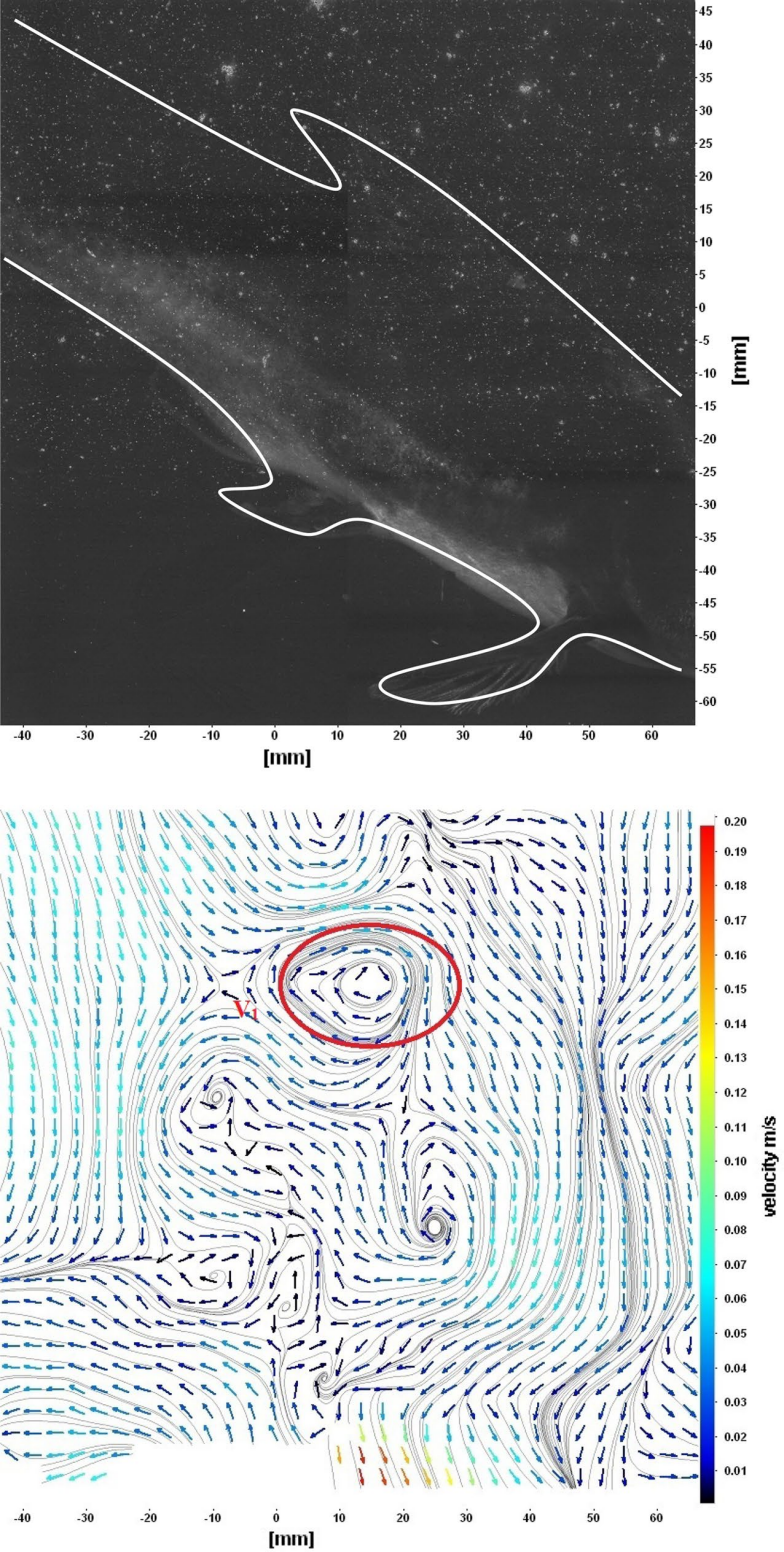
Raw image and velocity vector field around dorsal fins

**Fig. 14:**
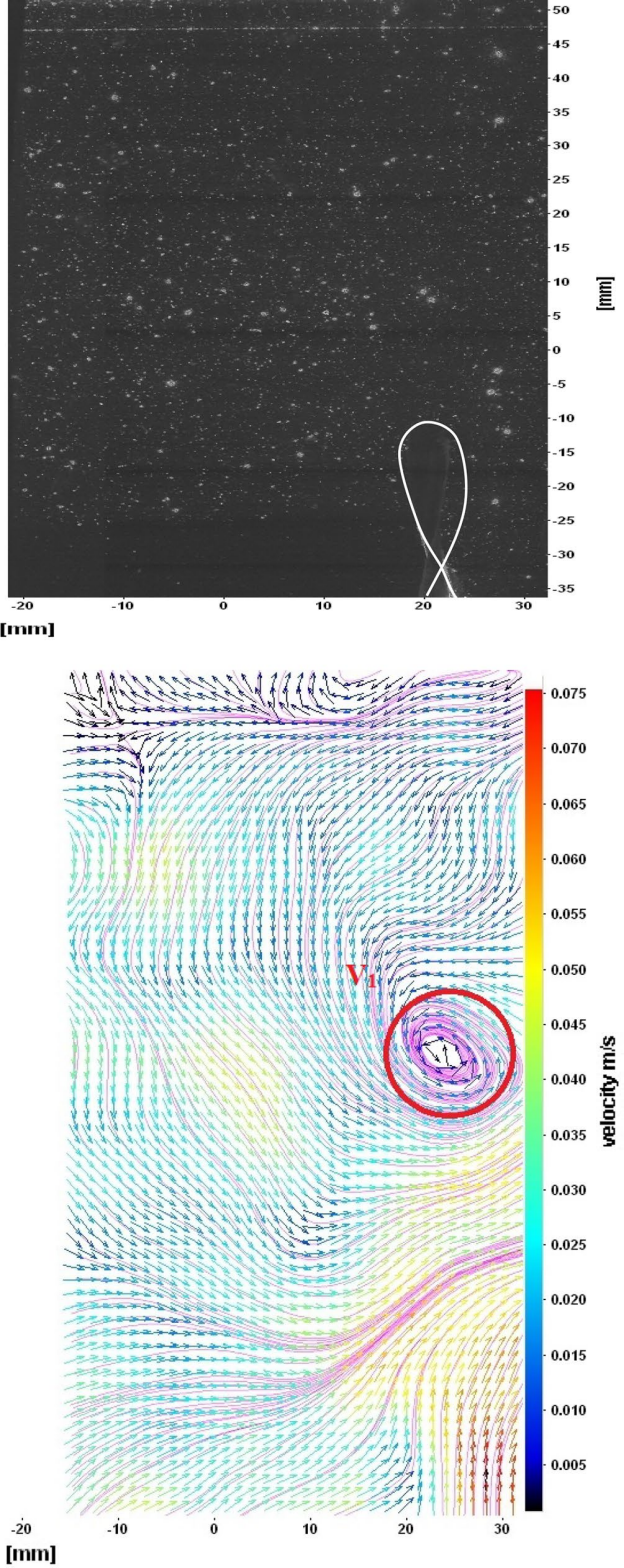
Raw image and velocity vector field around caudal fins starboard stroke in YZ plane at Δ*t* = 900 ms

**Fig. 15:**
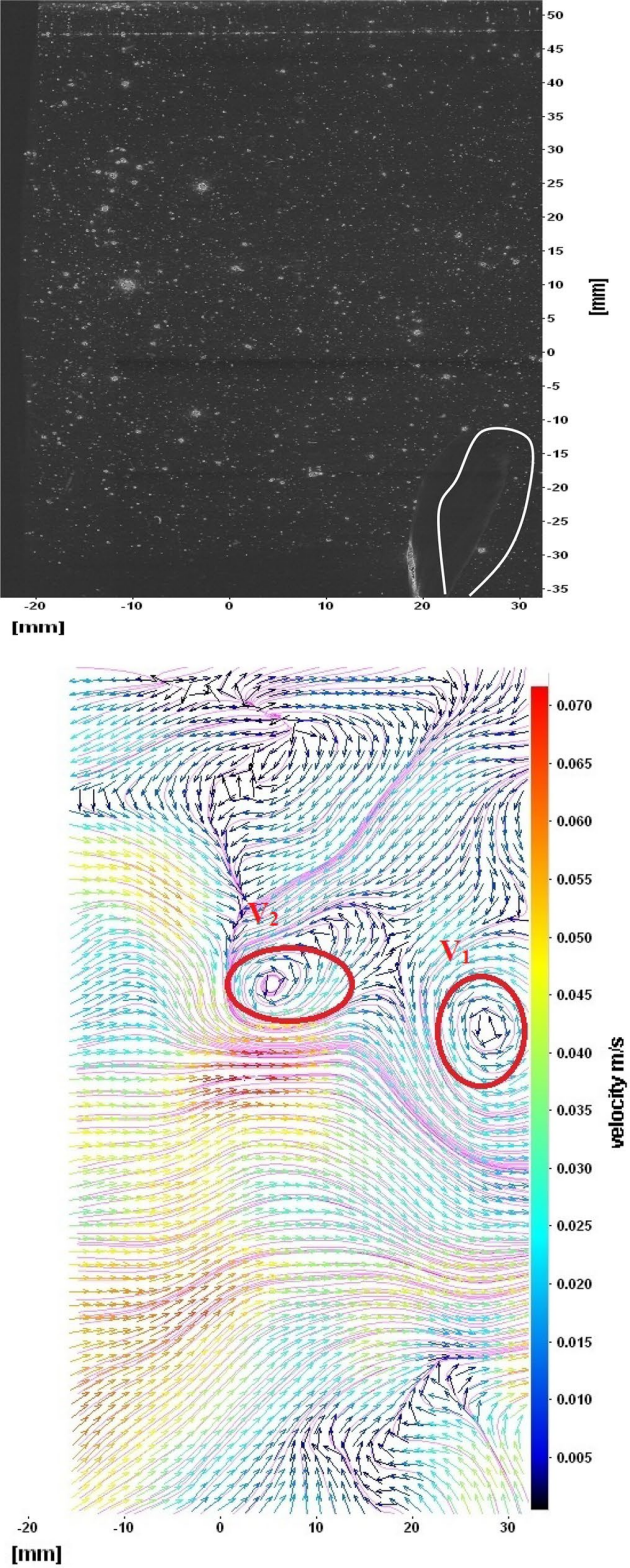
Consecutive raw image and velocity vector field around caudal fins during starboard stroke in YZ plane at Δ*t* = 900 ms

**Fig. 16:**
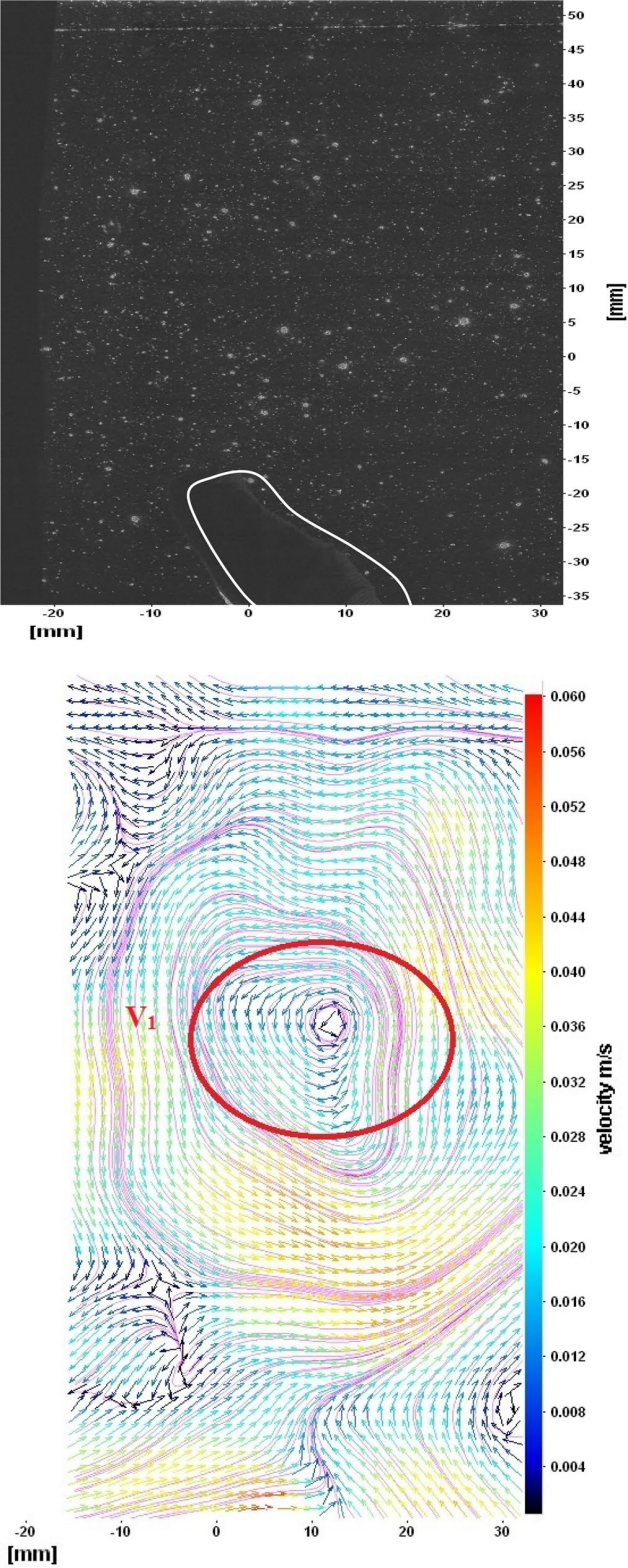
Consecutive raw image and velocity vector field around caudal fins stroke at center in YZ plane at Δ*t* = 900 ms

**Fig. 17:**
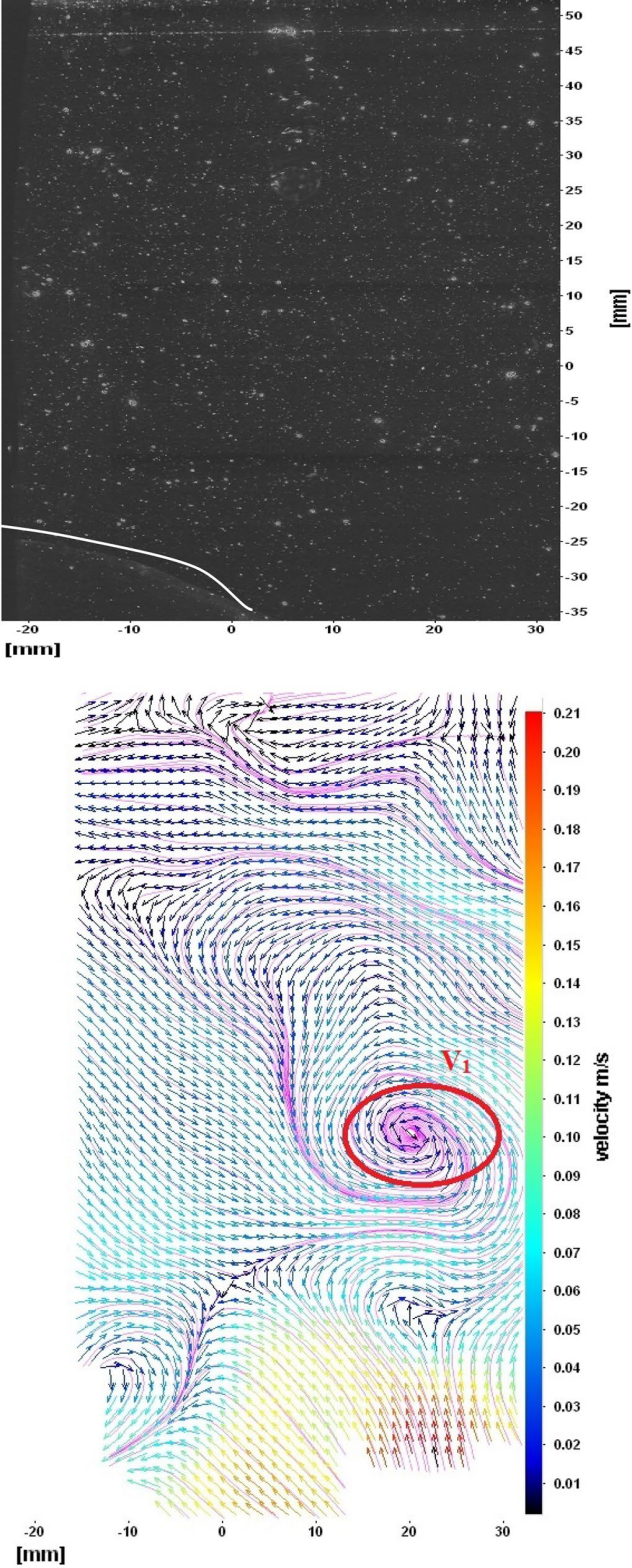
Consecutive raw image and velocity vector field around caudal fins during portside stroke in YZ Plane at Δ*t* = 900 ms

**Fig. 18:**
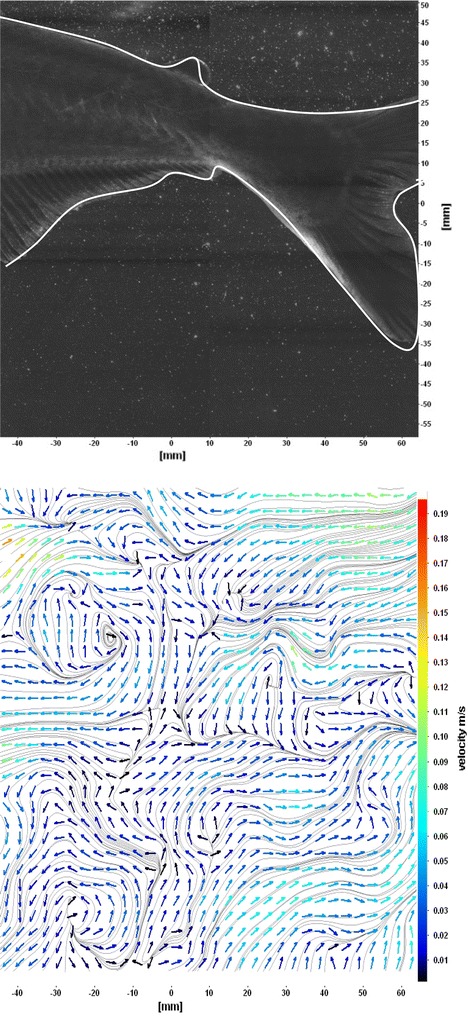
Flow around a maneuvering fish (tail end shown)

The flows around the fins of freely swimming fish are analyzed, and the velocity vector fields are presented here. In this analysis, we are presenting a raw CCD (charge-coupled device) image and the processed image at different time intervals (Figs. 7, 8, 9, 10, 11, 12, 13, 14, 15, 16, 17, 18). The white boundary line represents the body of fish. The primary vortex regions are marked as *V*_1_, *V*_2_ in images. Figure 7 shows CCD image and velocity vector field around caudal fin at Δ*t* = 900 ms. The caudal fin possess thrust-producing wake, resembling a reverse von Karman vortex street. In reverse von Karman vortex street, upper row vortices rotate in anticlockwise and the lower row vortices rotate in clockwise direction. Fishes are able to generate thrust depending on the amplitude and frequency of oscillation of the caudal fin. By varying the frequency and amplitude of fin oscillation, fishes can achieve the fin oscillation in the Strouhal number range of 0.2–0.5 to attain a propulsive force. The fish can move the caudal fin in both translational (sway) and rotational (yaw) modes for its efficient propulsion. The flow around the caudal fin of steadily swimming fish with counter-rotating vortices in vertical plane is shown in Fig. 7. The center of fish tail-shed vortices appears to be about 45 deg inclined to the centerline. During steady swimming, fishes orient the body at an angle to the flow. The propulsive force generated by caudal fin movement is directed to body through center of mass. Figures 8 and 9 show CCD image and velocity vector field around adipose and anal fins at Δ*t* = 900 ms. In the PIV experiments, the images are taken with a time difference between two consecutive images, Δ*t* = 300 and 900 ms. The image qualities are found to be acceptable in both the cases. The adipose and anal fins generated vortices that pass downstream, interacting with caudal fin vortices, while flapping its tail from starboard side to port side, and are found to form stronger vortices, thus helping in the generation of improved propulsive force.

Figure 10 shows CCD image and velocity vector field around adipose and anal fins at Δ*t* = 300 ms. Orientation of the caudal fin in this figure shows the flexibility present in its movements. The jets produced by adipose fin and anal fins are observed in the peduncle region (region containing the tail and the body). At the posterior end of the fish, a pair of counter-rotating vortices is observed. Figure 11 shows CCD image and velocity vector field around anterior portion of fish. At low amplitudes and frequencies of caudal fin, when Strouhal number (st) is less than 0.2, the vortices become inward and thus the fish experiences drag due to these vortices. This wake resembles like a von Karman vortex street shown in Fig. 11. Figure 12 shows CCD image and velocity vector field around pectoral fins. These paired pectoral fins undergo deformation during their flapping cycle. It undergoes chordwise and spanwise deformations as well as twisting. During power stroke and return stroke, the effective angle of attack of flow with fin increases, thereby producing thrust in both the strokes. Figure 13 shows CCD image and velocity vector field around dorsal fins. Dorsal fins generate strong vortices. Flow leaving the dorsal and anal fins rolls up and then interacts with caudal fin vortices. Figures 14 and 15 show CCD image and velocity vector fields around caudal fin in starboard stroke in YZ plane at Δ*t* = 900 ms. A pair of counter-rotating vortices is generated around the caudal fin in the YZ plane. Figure 16 shows CCD image and velocity vector field around caudal fin stroke in YZ plane at Δ*t* = 900 ms, while the fin is at the center plane. A jet with high velocity flow is observed at the top of the caudal fin. Figure 17 shows CCD image and velocity vector field around caudal fins, during portside stroke, in YZ plane at Δ*t* = 900 ms. Figure 18 shows flow around a maneuvering fish. During maneuvering of a fish, jets are observed at the side of fish causing a turning moment instantaneously.

## Summary and conclusions

The flow visualization experiments are carried out on a freely swimming freshwater black shark using two-dimensional particle image velocimetry in longitudinal vertical (XZ) and transverse (YZ) planes. The velocity vector fields show that both paired fins (pectoral fins) and median fins (dorsal, anal and caudal fins) produce reverse von Karman vortices resulting in the flow jets and consequent thrust (propulsive force). It is also observed that the fin flexibility in chordwise and spanwise direction substantially improves the thrust generation and direction control of the fish. The fish anal fin and caudal fin vortices are also presented here and show that they also contribute to the fish propulsive force. By studying the nature flow velocity distribution around fish fins propulsion systems, one can design flapping foil propulsion systems for ships and underwater vehicles.
